# Editorial: Advances and challenges in mobile robot design and control for diverse environments

**DOI:** 10.3389/frobt.2026.1816385

**Published:** 2026-03-09

**Authors:** Hongjun Xing, Weihua Li, Mojtaba Sharifi, Yuan Yang

**Affiliations:** 1 National Key Laboratory of Aerospace Mechanism, Nanjing University of Aeronautics and Astronautics, Nanjing, China; 2 School of Automotive Engineering, Harbin Institute of Technology, Weihai, China; 3 Department of Mechanical Engineering, San Jose State University, San Jose, CA, United States; 4 State Key Laboratory of Aerospace Flight Dynamics, Research Center for Intelligent Robotics, School of Astronautics, Northwestern Polytechnical University, Xi’an, China

**Keywords:** autonomous unmanned robots, kinematic and dynamic modeling, mechanism design, mobile robotic systems, motion planning and control

## 1 Introduction

In recent years, mobile robots have been increasingly deployed in diverse and unstructured environments, ranging from agricultural fields and confined spaces to human-centered indoor settings. Advances in nonlinear control, artificial intelligence, and innovative mechanical design have significantly expanded their autonomy and operational capabilities. However, mobile robot design and control in such environments still face substantial challenges, including nonlinear and nonholonomic dynamics, mapless navigation in unknown spaces, ensuring safety in human–robot coexistence, and the need to develop cost-effective but reliable system architectures.

Despite notable progress, many theoretical and practical issues remain unresolved. For instance, achieving robust trajectory tracking under model uncertainties, ensuring stable and efficient learning in sparse-reward scenarios, enabling controllable steering in soft and morphologically evolving robots, and integrating semantic information for safety-aware navigation all require further investigation. Therefore, in this Research Topic, we have collected recent contributions that address nonlinear control for agricultural robots, improved deep reinforcement learning for adaptive navigation, novel steering mechanisms for soft-growing robots, and multilayer safety frameworks for human-centered indoor environments. Together, these works provide timely insights into current developments and emerging challenges in mobile robotics.

## 2 Articles of this Research Topic

The articles contributing to the design and control of mobile robots for diverse environments included in this Research Topic are as follows:

Pulido-Aponte and Garzón-Castro focused on the control of mobile robots for agricultural inspection, specifically for soil resource monitoring in Solanum tuberosum crops. Their work presents an exact feedback linearization approach and a flat-filter-based controller to achieve robust trajectory tracking under nonholonomic constraints. Numerical simulations demonstrated that the proposed controller enables accurate navigation along linear and circular trajectories through crop rows, highlighting its potential for agro-industrial applications.

Nasti et al. proposed an improved deep reinforcement learning framework based on the Twin Delayed Deep Deterministic Policy Gradient (TD3) algorithm for adaptive mapless navigation in unknown environments. By integrating latent-state representation learning, intrinsic motivation, and regularization via maximum mean discrepancy, their approach reduces overestimation errors, enhances exploration efficiency, and stabilizes policy training. Experiments in ROS2/Gazebo simulations showed a high success rate and low collision frequency, demonstrating the framework’s effectiveness in safe and goal-directed navigation without prior maps.

Ji et al. presented a novel pivot-joint steering mechanism for tip-everting soft growing robots, addressing the challenge of directional control during continuous tip growth. The mechanism combines a tendon-driven pivot joint with a pressure-tunable internal bladder, enabling local bending and controlled forward growth without complex full-body actuation. Experimental validation confirmed reliable tip steering and close agreement with kinematic models, offering a scalable and structurally simple solution for navigation in confined spaces.

Omer and Monteriù developed a multi-layer robotic controller aimed at enhancing the safety of mobile robots operating in human-centered indoor environments. Their system combines an online human-in-the-loop layer, a semi-online layer that generates dynamic virtual barriers, and an offline layer that leverages semantic information from building digital twins. This architecture allows the robot to adapt to temporary hazards, user-defined constraints, and human presence while reducing reliance on complex real-time sensing, providing a cost-effective and safe navigation solution.

We believe this Research Topic of articles offers valuable insights into theoretical, algorithmic, mechanical, and system-level advancements in mobile robotics. It highlights current progress and emerging challenges in designing and controlling robots capable of operating safely and effectively across diverse environments.

